# Shall the Florence Nightingale Memorial Fail?

**Published:** 1911-10-21

**Authors:** 


### The Hospital

The Workers' Newspaper of Administrative Medicine and Institutional Life, Administration, National Insurance and Health.

No. 1319, Vol. LI. SATURDAY, OCTOBER 21st, 1911. PRICE ONE PENNY.

### SHALL THE FLORENCE NIGHTINGALE MEMORIAL FAIL?

The saddest experience of recent times must be surely to promote a memorial to the dead which proves a failure and threatens dishonour to the memory of the great departed. A valuable life like Florence Nightingale's passes. The peoples of the world owe more perhaps to her than to almost any woman that ever lived, and yet we are face to face with the lamentable fact that the sweetness and perfection of her record at the date of her death may suffer diminution through want of general interest.

At a meeting of the general committee of the Florence Nightingale Memorial held at St. Thomas's Hospital on October 17 it was reported that of £5,000 required a nett sum of £2,500 had been subscribed, most of which was available for the statue proposed to be erected on a suitable site in London. Yet Florence Nightingale's work and the inspiration it gave to all workers among the sick should awaken something better than gratitude, for it constituted a living force which has influenced the lives of hundreds of thousands among us to their infinite uplifting and benefit. An inspection of the sources from which the small sum which has so far been received for her memorial shows that a fifth of it has come from a relatively few nurses mostly connected with less than a dozen institutions. The soldiers of the British Army, to their honour, with the sailors, too, have done their part, but the public and the individual worker, so far as this subscription list is concerned, are conspicuous by their absence. Why is this? Is it because the present generation is not conscious of the infinite debt which it owes to Florence Nightingale's teaching and spirit? We cannot credit it. Think of it, every worker among the sick, whoever and wherever you may be. The small sum of £5,000 was asked for to defray the cost of a statue worthy of her memorial and not half of the money is forthcoming though over a year has elapsed since she died!

Our faith is so strong in the reality of the spirit with which she has inspired every worker and many thinking men and women of the British race, that we have decided to place the facts thus frankly before all whom it may concern and to invite their individual co-operation without a moment's delay. A further £2,500 is required to complete the memorial and make it worthy of the great woman whose memory it will perpetuate in the years that are to come. We want some seventy-five men and women of spirit and energy to send us their names at once, or the names of the institution they represent, with an undertaking that fifty of them will throw themselves heart and soul into this movement and by personal service each raise a sum of £25, and twenty-five others who will by personal service make themselves responsible for £50 each. It is not an occasion when some one or two rich people can be permitted to give of their abundance in order to save the British people from the national disgrace with which they are threatened by a failure of the Florence Nightingale Memorial Fund. Her memory is in the custody and care of all workers for the sick who feel what they owe to Florence Nightingale's inspiration and work. Theirs are the responsibility and privilege to give of themselves promptly and make Florence Nightingale's Memorial worthy of her life's work.

We do not believe in asking others to do something which we are not prepared to do ourselves. We have, therefore, decided to embrace the opportunity to be one of the fifty who provide £25 each, and our staff, being imbued with the same sentiments, has at once claimed the right to be among the twenty-five contributors of £50. Do not let any man or woman in a position to do so hesitate to send us his or her name at once and to set to work to complete this matter without a moment's avoidable delay. Ours is the privilege; let us, now that we know the facts, show that the spirit with which we enter into our work for the sick is a real living force, a treasured possession which will never let us rest until the Florence Nightingale Memorial is completed and her memory worthily commemorated. Who amongst us can consent to permit the work of Florence Nightingale to be belittled by failure when she is worthy of the very best memorial which human accomplishment, skill, knowledge, love and tender care can provide? Are there not fifty institutions where at least one leading worker in each will make the main facts known and receive £50 or £25 as circumstances may permit? Who can hesitate to lend a hand? Recall the Lady with the Lamp; may memory, the Council chamber of thought, awaken, and so equipped let us go forth with gratitude in our hearts. Let us each do our part promptly with the zeal of conviction by the devotion of all our energies to the purpose until it is fulfilled!

